# Supplementary material for: Patterns of Intron Gain and Loss in Fungi
Source: PLoS Biol. 2004 Nov 30;2(12):e422. doi: 10.1371/journal.pbio.0020422 (PMC532390; doi:10.1371/journal.pbio.0020422)
Supplement: Table S1 — Also available at http://genes.mit.edu/NielsenEtAl/. (4.3 MB ZIP). [file pbio.0020422.st001.zip › NielsenEtAl/html/1198.html]

AN4050.1.NCU02056.1.MG00652.1.FG00718.1


```
 CLUSTAL W (1.82) Multiple Sequence Alignments - Introns Inserted


Sequence 1: NCU02056.1	476 aa
Sequence 2: FG00718.1	477 aa
Sequence 3: MG00652.1	479 aa
Sequence 4: AN4050.1	482 aa
Alignment Length: 483 aa
Number Identitical Residues: 182 aa
Alignment Score (without introns) 10184


MG00652.1 	----MGGEKDFIVPLVIGGKEKTTSDSFPVVSPATGETVHKCSNANVADAEEAVEAAAAA
NCU02056.1	------MAETYTVPFFINGEEVRTSRTFDVTSPATQKVVHKCSSATDTEVQAAVNAAAEA
FG00718.1 	-----MSAKLHTVPFLINGSDHVSETTADVVSPVNGEVTHRYSSADVKDANSAVDAAAEA
AN4050.1  	MASNHTNGDKAMLPLIINNESVITDNVVEVHNPATGELLHRCAGASVDDANRAVAAAKAA
          	 ::.    .   :*:.*....  :.    * .*.. :  *: :.*   :.: ** **  *

MG00652.1 	LNDWKRKTPAERRDIFLKAAQILVQRTDELASYMESETAASRNWANFNLDLTREMLIDVA
NCU02056.1	FKTWRKSHPNQRRDLFHKAAEILEKRKEELGQYVMDETGAPRQWADFNIQVAKDLLMDVA
FG00718.1 	FKSWRKTRPSERRDLLLKVAEIMERRQEELRGYAMTECGSDAAWASFDVNTGISHIKEIA
AN4050.1  	FPIWSKTHPYERRAILSKAADIMFSRKEEFIKTQMEETGAGRMFVEVTFMASVSFLRDFA
          	:  * :. * :** :: *.*:*:  * :*:      * .:   :... .    . : :.*

MG00652.1 	GRISAATTGAIPATRDANLSAMVVKEPYGVVLAMAPW~NAPYILGMRAVLFPLAVGNTVV
NCU02056.1	GRCN-TLEGSFPVVNDPESGALVLQEPYGVILAVAPW2NAPYILGIRSILYPIAAGNTAI
FG00718.1 	GRVG-TIEGSIPTVADPNTTALVLREPYGVVVAIAPW2NAPYILGTRSVLFPIAAGNTVV
AN4050.1  	GMIP-SVEGRAPIVAEEGQSALVIKQPYGVVLGIAPW2NAPFILGTRSVALPLAAGNTTI
          	*    :  *  * . :    *:*:::****::.:*** ***:*** *::  *:*.***.:

MG00652.1 	FKGSELSPRTMWGICSVLAEAGVPHGALSLIFCSRETAASVTETLIAHKHMKKINFTGST
NCU02056.1	FKATELCPRTMWGLCSVFHEAGLPKGVLNMLVHEPANAPAITSSLISNPQVKKVNFTGST
FG00718.1 	FKASETCPRTFWAIGDIFREAGFPDGVLNVIYHERANAASVTTALIEHPQVKKINFTGST
AN4050.1  	LKGSELSPKCFWLIGDVLREAGLPAGCLNVIYHKTSDAPAVTNALIAHPDVRKISFTGST
          	:*.:* .*: :* : .:: ***.* * *.::  .   *.::* :** : .::*:.*****

MG00652.1 	SVGRIIGRLSGQHLKPVLLELGGKAPAIVWEDADLDNAAAQCTLGAYLA~AGQVCMSTER
NCU02056.1	AVGRIIGRLAGENLKPVLLELGGKASAIVWEDAELDNAATQCALGAFLN~SGQICMSTER
FG00718.1 	PVGRLIGKIAGENLKPVILELGGKAPAIVWEDADLDLAALQCTLGAFIN~SGQVCMSTER
AN4050.1  	LVGSIIASTAGKYIKPVLLELGGKASAIVLDDADLEKAAMGCTLGAFLN0SGQICMSTER
          	 ** :*.  :*: :***:*******.*** :**:*: **  *:***::  :**:******

MG00652.1 	IIVHKAVSEQFRGKFAACVDKFFPSSADAPILVQSQGVTRNHELLKDALSKGAEVVVGDA
NCU02056.1	ILVHKSVRAEFEKKLVRAVENIFGPPTPAPILINSLAVDKNKKLLSDALSKGAKLLYGNP
FG00718.1 	ILVHKNIKDEFEKKLSATIDQVFSSAADAPILVASAPVEKNKALIKDAIAKGASLAHGDV
AN4050.1  	IVVQRPVAEKFQKLLVETSEKIFGKNAPAPVLVATAAVKKNQGLVADAIDKGASVVFGDP
          	*:*:: :  :*.  :    ::.*   : **:*: :  * :*: *: **: ***.:  *: 

MG00652.1 	EAKEANAHSMRPVAISGVTPDMDIYMTESFGPTVSLIEVSSEEEALRIANDTEYGLSSAV
NCU02056.1	DAQEETTTRMRPVIVSGITTDMDIYKTESFGPTVAVYEIETEEEALRIANDTEYGLTSAV
FG00718.1 	DAKEVSNTRMRPIVVRDVTAEMDIYKTESFGPTVSLISIETEEEAIKIANDTEYGLSSAV
AN4050.1  	KESEPCANALRPVIVGGVTKEMDLYATESFGPTVSLIVVDSEEEAIKVANDTEYGLTSAV
          	. .*     :**: : .:* :**:* ********::  :.:****:::********:***

MG00652.1 	FTADLRRALRLAHGIETG~AVHINSMSVHDETGLPHGGAKASGFGRFNTADGLNEWLRTK
NCU02056.1	FTEDLRRGLRMAKEIETG~AVHINSMTVHDEATLPHGGAKASGYGRFNTSRGLQEWVKSK
FG00718.1 	FTSDLQRGLRIAREIESG~AVHINNMSVHDESGLPHGGAKSSGYGRFGTSAGLEEWTRTK
AN4050.1  	FTSNLFRGLRVAKQIESG2AVHINSMTVHDEPTLPHGGWKSSGFGRFGGTAGYDEWLQTK
          	** :* *.**:*: **:* *****.*:****. ***** *:**:***. : * :** ::*

MG00652.1 	NITYRI
NCU02056.1	TVTFKF
FG00718.1 	NITFKN
AN4050.1  	TITWVE
          	.:*:
```
